# Combined blockade of GPX4 and activated EGFR/HER3 bypass pathways inhibits the development of ALK‐inhibitor‐induced tolerant persister cells in *ALK* ‐fusion‐positive lung cancer

**DOI:** 10.1002/1878-0261.13746

**Published:** 2024-10-06

**Authors:** Koh Furugaki, Takaaki Fujimura, Narumi Sakaguchi, Yasutaka Watanabe, Ken Uchibori, Eisaku Miyauchi, Hidetoshi Hayashi, Ryohei Katayama, Shigeki Yoshiura

**Affiliations:** ^1^ Product Research Department Chugai Pharmaceutical Co., Ltd Yokohama Japan; ^2^ Biological Technology Department Chugai Pharmaceutical Co., Ltd Yokohama Japan; ^3^ Division of Thoracic Oncology Saitama Cancer Center Saitama Japan; ^4^ Department of Thoracic Medical Oncology The Cancer Institute Hospital, Japanese Foundation for Cancer Research Tokyo Japan; ^5^ Department of Respiratory Medicine Tohoku University Graduate School of Medicine Sendai Japan; ^6^ Department of Medical Oncology Kindai University Faculty of Medicine Sayama Japan; ^7^ Division of Experimental Chemotherapy Cancer Chemotherapy Center, Japanese Foundation for Cancer Research Tokyo Japan

**Keywords:** ALK, drug‐tolerant persister, EGFR, FGFR, GPX4

## Abstract

Cancers can develop resistance to treatment with ALK tyrosine kinase inhibitors (ALK‐TKIs) via emergence of a subpopulation of drug‐tolerant persister (DTP) cells that can survive initial drug treatment long enough to acquire genetic aberrations. DTP cells are thus a potential therapeutic target. We generated alectinib‐induced DTP cells from a patient‐derived *ALK* ^+^ non‐small‐cell lung cancer (NSCLC) cell line and screened 3114 agents in the anticancer compounds library (TargetMol). We identified phospholipid hydroperoxide glutathione peroxidase GPX4 as being involved in promoting the survival of DTP cells. GPX4 was found to be upregulated in DTP cells and to promote cell survival by suppressing reactive oxygen species (ROS) accumulation; GPX4 inhibitors blocked this upregulation and facilitated ROS‐mediated cell death. Activated bypass signals involving epidermal growth factor receptor (EGFR)/receptor tyrosine‐protein kinase erbB‐3 (HER3) were also identified in DTP cells, and co‐treatment with EGFR‐TKI plus ALK‐TKI enhanced ROS levels. Triple combination with an ALK‐TKI plus a bypass pathway inhibitor plus a GPX4 inhibitor suppressed cell growth and induced intracellular ROS accumulation more greatly than did treatment with each agent alone. The combined inhibition of ALK plus inhibition of activated bypass signals plus inhibition of GPX4 may be a potent therapeutic strategy for patients with *ALK* ^+^ NSCLC to prevent the development of resistance to ALK‐TKIs and lead to tumor eradication.

## Graphical abstract

ALK inhibitor treatment of *ALK‐*fusion‐positive non‐small cell lung cancer cells leads to drug‐tolerant persister (DTP) cells with increased reactive oxygen species (ROS). DTP cells upregulate GPX4 protein expression to evade ROS‐mediated cell death and activate EGFR/HER3 bypass signaling. Triple combined inhibition with ALK, GPX4 and EGFR/HER3 suppresses DTP cell survival, providing a potential strategy to prevent ALK inhibitor resistance.

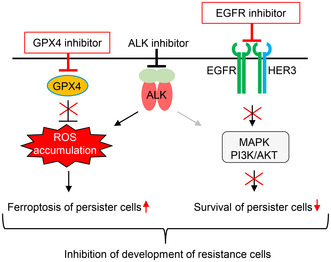

**Abbreviations**
ALCL: anaplastic large‐cell lymphoma
ALCURE: ALeCtinib Ultimate REsistance mechanisms study
ALK: anaplastic lymphoma kinase
*ALK* ^+^
: ALK‐fusion‐positive
AP‐1: activator protein 1
CAFs: cancer‐associated fibroblasts
DEGs: differentially expressed genes
DMSO: dimethyl sulfoxide
DTP: drug‐tolerant persister/persistence
FBS: fetal bovine serum
FTH1: ferritin heavy chain 1
GPX4: glutathione peroxidase 4
GSEA: Gene Set Enrichment Analysis
GSH: reduced glutathione
GSK3: glycogen synthase kinase 3
GSSG: oxidized glutathione
JNK: c‐Jun N‐terminal kinase
NRG1: neuregulin 1
NSCLC: non‐small‐cell lung cancer
ROS: reactive oxygen species
TKI: tyrosine kinase inhibitor

## 1 Introduction

ALK‐fusion‐positive non‐small‐cell lung cancer (*ALK* ^+^ NSCLC) is a distinct molecular subtype of NSCLC characterized by a rearrangement of the anaplastic lymphoma kinase (*ALK*) gene [1]. Patients with the *ALK* ^+^ NSCLC subtype have been found to have improved clinical outcomes when treated with ALK tyrosine kinase inhibitors (ALK‐TKIs), which has led to the approval of agents such as crizotinib, alectinib, ceritinib, brigatinib, and lorlatinib [2]. However, despite the initially high rates of response to these ALK‐TKIs, resistant tumor cells can develop due to secondary mutations in the ALK tyrosine kinase domain, activation of bypass signaling pathways or other alterations that cause phenotypic changes, loss of target, or loss of target dependency [3]. One process by which drug resistance is thought to be acquired is via the entry of a small population of cancer cells into a reversible, slow‐growing state of “drug‐tolerant persistence” [4]; these drug‐tolerant persister (DTP) cells evade cell death induced by targeted therapy long enough to acquire multiple additional and complex resistance mechanisms with genetic aberrations [5].

Despite the accumulating *in vitro* evidence for the presence of DTP cells [5], the mechanisms by which DTP cells modulate their tolerance to targeted TKI therapy remain poorly understood. Although more efficacious targeted therapies should be implemented up‐front to prevent the emergence of DTP cells, in order to achieve this aim, it is essential to identify the mechanisms by which a targeted ALK‐TKI–induced DTP state is acquired and to select a suitable therapeutic strategy that suppresses the generation of DTP cells.

In this study, we screened an anticancer compound library with *ALK* ^+^ NSCLC cells derived from an ALK‐TKI treatment‐naïve patient and identified glutathione peroxidase 4 (GPX4) as a potential candidate that could be targeted to suppress alectinib‐induced DTP cells.

GPX4 is a selenoenzyme that catalyzes the reduction of lipid‐based reactive oxygen species (ROS), particularly lipid hydroperoxides, to the corresponding alcohols or water, by converting reduced glutathione (GSH) into oxidized glutathione (GSSG) [6]. Because GSH is one of the major scavengers of ROS, the ratio of GSH to GSSG mirrors the levels of ROS; accordingly, the GSH/GSSG ratio is a useful marker of ROS‐mediated oxidative damage, with a low GSH/GSSG ratio being a sign of oxidative stress [7]. GPX4 is also a key suppressor of ferroptosis, an iron‐dependent type of programmed cell death. Ferroptosis is induced when the iron‐storage protein ferritin and the ferritin heavy chain 1 (FTH1) increases intracellular free iron levels, leading to a lethal accumulation of intracellular ROS [8]. Owing to their high levels of polyunsaturated fatty acids, cellular membranes are especially susceptible to ROS‐mediated oxidative damage, which is called lipid peroxidation [7]. Within this process, GPX4 inhibits the formation of lipid peroxides and plays a pivotal role in the avoidance of ROS‐induced ferroptosis [9].

Recently, Hangauer et al. [10] reported that GPX4 inhibition has potent activity to suppress survival of DTP cells, including those generated from HER2‐amplified breast cancer, EGFR‐mutated lung cancer, and BRAF‐mutated melanoma cells. However, it is unknown whether GPX4 inhibition is sufficient to prevent the emergence and survival of DTP cells in *ALK* ^+^ NSCLC cells.

In this study, we explored the effects of GPX4 inhibition on the emergence and survival of DTP cells in *ALK* ^+^ NSCLC cells. Additionally, we tried to develop a novel combinational therapeutic strategy for overcoming acquired ALK‐TKI resistance via inhibiting generation of DTP cells.

## 2 Materials and methods

### 2.1 Cell lines

This study was conducted in accordance with the Declaration of Helsinki in Japan. A patient‐derived *ALK* ^+^ cell line was established from an ALK‐TKI treatment‐naïve patient with *ALK* ^+^ NSCLC who had provided informed consent for genetic and cell biological analyses in the ALCURE (ALeCtinib Ultimate REsistance mechanisms) study (UMIN000038934). The study protocol of ALCURE was reviewed and approved by the institutional review board from the perspective of ethical, scientific, and medical validity in Japanese Foundation For Cancer Research and Chugai Pharmaceutical Co. Ltd (E19081). All patients provided written informed consent prior to any study‐related procedures. A single clone of these *ALK* ^+^ cells, designated ALK1903 cells, was used in this study. ALK1903 cells were established at the Japanese Foundation For Cancer Research from tumor tissue samples collected in the Division of Thoracic Oncology at Saitama Cancer Center in Saitama, Japan, in February 2020. ALK1903 cells express EML4‐ALK variant 3a. The cells were maintained in StemPro hESC SFM (Thermo Fisher Scientific, Waltham, MA, USA), and cultured in equal proportions of RPMI‐1640 medium (Sigma‐Aldrich, St. Louis, MO, USA) and Ham's F‐12 medium (Fujifilm Wako Pure Chemical Corporation, Osaka, Japan) supplemented with 15% fetal bovine serum (FBS; Sigma‐Aldrich) and 20 mM HEPES (Nacalai Tesque Inc., Kyoto, Japan) from 1 week before DTP cell preparation. NCI‐H2228 lung cancer cells (RRID: CVCL_1543) were purchased from the American Type Culture Collection (ATCC) and were maintained in RPMI‐1640 medium (Sigma‐Aldrich) supplemented with 10% FBS. NCI‐H2228 cells were isolated in 1989 from a lung adenocarcinoma derived from a female nonsmoker with non‐small cell lung cancer at the National Cancer Institute (NCI) according to Institutional Review Board‐approved clinical protocols after the patient signed a consent form to allow her tissue for cell line growth [11]. NCI‐H2228 cells have variant 3a of the EML4‐ALK fusion gene, and their cell growth depends on the ALK signaling pathway [12]. All cells were maintained at 37 °C under 5% CO_2_ under the mycoplasma‐free conditions. All cancer cell lines were authenticated by short tandem repeat (STR) DNA profiling.

### 2.2 Drugs and reagents

Alectinib was synthesized by Chugai Pharmaceutical Co., Ltd. (Tokyo, Japan). Erlotinib was provided by F. Hoffman‐La Roche Ltd. (Basel, Switzerland). Infrigatinib (BGJ398) was obtained from LC Laboratories (Woburn, MA, USA). Lorlatinib, RSL3, ML210, erastin, and ferrostatin‐1 were obtained from Selleck Chemicals (Houston, TX, USA). N‐acetyl cysteine was obtained from NACALAI TESQUE Inc. The anticancer compound library was obtained from TargetMol (Boston, MA, USA). N‐acetyl cysteine was dissolved in water; all other agents were dissolved in dimethyl sulfoxide (DMSO; Sigma‐Aldrich).

### 2.3 Anticancer compound library screen of ALK1903 cells

ALK1903 parental and DTP cells were seeded in 384‐well plates, and the 3114 agents in the anticancer compound library were added at 100 nm on the following day. After 6 days, cell viability was determined by quantitation of cellular ATP, which indicates metabolically active cells, using a CellTiter‐Glo 3D Cell Viability Assay kit (Promega, Madison, WI, USA). Viability with the agents relative to viability with the vehicle was measured, and the survival ratio of DTP cells with respect to parental cells was calculated for each agent as follows: (viability of DTP cells)/(viability of parental cells).

### 2.4 Cell proliferation assay

Cells were seeded in 384‐well plates, and drugs were added at the indicated concentrations the following day. After 6 days, cell viability was determined using the CellTiter‐Glo 3D Cell Viability Assay. Viability with the agents relative to viability with the vehicle was measured. Each point represents the mean ± standard deviation of triplicate experiments. IC_50_, IC_40_, IC_30_, and IC_20_ values were calculated as described previously [13].

### 2.5 Western blotting

Cells were seeded in 6‐well plates and drugs were added at the indicated concentrations the following day and cultured for the indicated time. The same amount of protein lysate was loaded for each western blotting assay using a Jess capillary electrophoresis‐based protein analysis system (ProteinSimple, San Jose, CA, USA) according to the manufacturer's protocol. Antibodies against ALK, phospho‐ALK, ERK, phospho‐ERK, AKT, phospho‐AKT, EGFR, phospho‐EGFR, HER3, phospho‐HER3, HER2, CD44, CD133, BIM, VIM, CDH1, STAT3, phospho‐STAT3, GPX4, CD98, FTH1, NRG1 and β‐actin (Cell Signaling Technology, Danvers, MA, USA), cleaved PARP, phospho‐HER2, and xCT (Abcam, Cambridge, UK) were used. The supplier, catalog number, and phospho‐site number of each antibody is shown in Table S1.

### 2.6 Apoptosis assay

Cells were seeded in 384‐well plates, and the following day, drugs were added at the indicated concentrations and cultured for 2 days. The activities of caspase‐3 and ‐7 were determined by using the Caspase‐Glo 3/7 Assay (Promega). The activity was corrected with respect to cell viability determined as described above, and activity with the agent relative to activity with the vehicle was calculated. Each point represents the mean + standard deviation of triplicate experiments.

### 2.7 ROS measurement

After washing the cells with PBS, cells were cultured for 30 min at 37 °C in 5‐(and‐6)‐chloromethyl‐2′,7′‐dichlorodihydrofluorescein diacetate, acetyl ester (CM‐H_2_DCFDA; Thermo Fisher Scientific) to stain ROS, followed by 15 min incubation with Hoechst‐33 342 (M&S TechnoSystems, Osaka, Japan) to determine the position of cells. The fluorescence intensity of CM‐H2DCFDA per cell, which indicates the level of cellular ROS, was measured and analyzed with an NC‐3000 cell counter (M&S TechnoSystems).

### 2.8 Lipid peroxide measurement

Following the same protocol as described for ROS measurement, cells were cultured for 30 min in Lipid Peroxidation Probe (Dojindo, Kumamoto, Japan) to stain lipid peroxide, followed by incubation with Hoechst‐33342. The fluorescence intensity per cell, which indicates the level of cellular lipid peroxide, was measured and analyzed with an NC‐3000 cell counter.

### 2.9 GSH/GSSG measurement

After washing the cells with PBS, the GSH/GSSG level was measured with GSH/GSSG‐Glo Assay (Promega).

### 2.10 RNA‐seq analysis

As for RNA‐seq analysis of ALK1903 cells, total RNA was obtained from ALK1903 or its DTP cells with or without 1000 nm alectinib treatment for 9 days using the Maxwell RSC simplyRNA Tissue Kit (Promega). Transcriptome libraries were prepared using the TruSeq Stranded mRNA Library Preparation Kit (Illumina, San Diego, CA, USA), and next‐generation sequencing was performed on a NovaSeq6000 system (Illumina) with paired‐end 150 bp reads at Macrogen Japan Corp (Tokyo, Japan). After excluding read pairs with a mapping quality of < 20 using Trimmomatic, the sequence reads were aligned to the Hg38 reference genome using STAR, and read count data obtained with prepDE.py. Differentially expressed genes of antioxidant genes were analyzed by the enrichment analysis using single‐sample gene set enrichment analysis (ssGSEA) with GSEApy library ver. 1.0.5. Heatmap was generated using Z score calculated by logarithmic conversion of TPM data using SciPy library ver. 1.10.1.

### 2.11 NGS and copy number analysis

Genomic DNA was obtained from ALK1903 cells using the Maxwell 16 Tissue DNA purification Kit (Promega). The Thermo Fisher Scientific Oncomine™ Pan‐Cancer Cell‐Free Assay was used to examine the somatic mutations, and copy number variations of cancer‐related genes and major fusion oncogenes, such as TP53, EGFR, BRAF, KRAS or PDGFRA mutations, and ALK, ROS1, or RET fusions and so on. After purify the genomic DNA and total RNA from ALK1903 cells, NGS was conducted according to the manufacture's protocols and as previously reported [14]. Sequencing data was mapped to hg38 and analyzed using on‐instrument software. Detected alterations were annotated using Oncomine Knowledgebase Reporter Software (Oncomine Reporter 5.0). Copy numbers of DNA were measured using the predesigned TaqMan copy number probe for human *ALK* (Thermo Fisher Scientific, 4 400 291) with quantitative real‐time PCR using a LightCycler 480 System (Roche Diagnostics, Basel, Switzerland). Copy numbers normalized to the reference control gene of human RNase P (Thermo Fisher Scientific) were analyzed using copycaller ver. 2.1 software (Thermo Fisher Scientific) with human genomic DNA (Promega) as diploid control DNA.

### 2.12 Statistical analysis

Experimental data were analyzed by Student's *t*‐test or Dunnett's test followed by the Holm method. All statistical analyses were conducted using JMP Ver. 15.0.0 (SAS Institute, Cary, NC, USA). Significance was set at a two‐tailed *P* < 0.05.

## 3 Results

### 3.1 Alectinib treatment generates DTP cells from patient‐derived ALK1903 cells

**Fig. 1:**
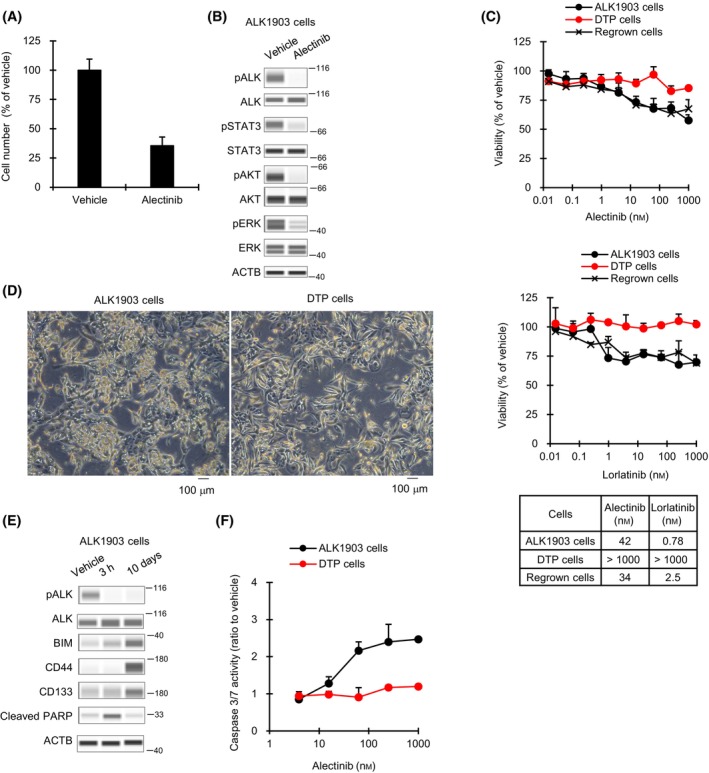
Effect of ALK inhibitors on ALK1903, DTP, and regrown cells. (A) Cells were cultured with 1000 nm alectinib for 2 weeks (*n* = 3). (B) Immunoblots of cell lysates from ALK1903 cells treated with 1000 nm alectinib for 3 h (*n* = 1). The data represented an image from two independent experiments. (C) After establishment of DTP (drug‐tolerant persister) cells, the cells were regrown without alectinib for 77 days. Parental, DTP, and regrown cells were each cultured with alectinib or lorlatinib for 6 days (*n* = 3). The IC_30_ values for alectinib and IC_20_ values for lorlatinib are shown. (D) Microscopic examination of ALK1903 parental and DTP cells generated from ALK1903 cells by treatment with 1000 nm alectinib for 9 days (*n* = 1). Scale bars indicate 100 μm. The data represented an image from two independent experiments. (E) Immunoblots of cell lysates from ALK1903 cells treated with 1000 nm alectinib for 3 h or 10 days (*n* = 1). The data represented an image from two independent experiments. (F) ALK1903 and DTP cells were cultured with alectinib for 2 days, and caspase 3/7 activities were measured (*n* = 3). Values were shown as mean ± SD (standard deviation). Data are representative of two independent experiments.

To find novel targets that promote cell survival in *ALK* ^+^ NSCLC cells subjected to ALK inhibition, we first established a cell line (ALK1903 cells) with cells from an ALK‐TKI treatment‐naïve *ALK* ^+^ NSCLC patient. In ALK1903 cells, alectinib inhibited cell proliferation and the phosphorylation of ALK, and also inhibited the phosphorylation of STAT3, AKT, and ERK, which are involved in ALK downstream signaling (Fig. 1A,B). Alectinib and lorlatinib each inhibited cell viability in a dose‐dependent manner (Fig. 1C). We additionally performed a western blot with antibodies to detect phospho‐ALK (Y1278), phospho‐ALK (Y1604), and total ALK with an increasing amount of alectinib, from 0.01 to 1000 nm, for 3 h. The phosphorylation levels of ALK of Y1278 and Y1604 as well as STAT3, AKT, and ERK were inhibited by 100 and 1000 nm of alectinib treatment (Fig. S1). Under the long‐term exposure conditions for 12 days, the IC_50_ values of alectinib and lorlatinib were 100.4 and 2.8 nm, respectively.

We performed NGS analysis with an Oncomine assay, which detects hot spot mutations, fusions, *MET* exon 14 skipping, and the copy numbers of 52 genes including major oncogenes such as *EGFR*, *ERBB2*, *ERBB3*, *KRAS*, and *BRAF*. The NGS analysis showed only *EML4‐ALK* variant 3 and S478P of *platelet‐derived growth factor receptor alpha* (*PDGFRA*) in ALK1903 cells, ant the clinical significance of *PDGFRA* (S478P) mutation is benign in the ClinVar database (https://www.ncbi.nlm.nih.gov/clinvar/). The copy number of *ALK* in ALK1903 cells was 2.6 ± 0.7.

Then, we generated DTP cells from ALK1903 cells that survived 9 days of 1000 nm alectinib treatment. The alectinib‐induced ALK1903 DTP cells showed features common to DTP cells, such as morphological alterations (Fig. 1D) and upregulation of stemness marker proteins CD44 and CD133 (Fig. 1E) [5, 15]. Moreover, apoptosis, which is mirrored by the amount of cleaved PARP protein, was immediately induced in ALK1903 cells via suppression of ALK signaling after 3 h of alectinib treatment, whereas induction of apoptosis had disappeared after treatment with alectinib for 10 days (Fig. 1E). In line with western blot analysis, caspase 3/7 assay showed that apoptosis was not induced in ALK1903 DTP cells by alectinib as compared to in parental ALK1903 cells (Fig. 1F). On the other hand, regrown cells generated from DTP cells by culturing in alectinib‐free medium for 77 days, showed restored sensitivity to ALK‐TKIs alectinib and lorlatinib (Fig. 1C). These results indicate that ALK1903 cells survived by promoting a DTP state in the presence of alectinib.

### 3.2 Compound screening identifies GPX4 as a candidate promoting alectinib‐induced DTP cells

**Fig. 2:**
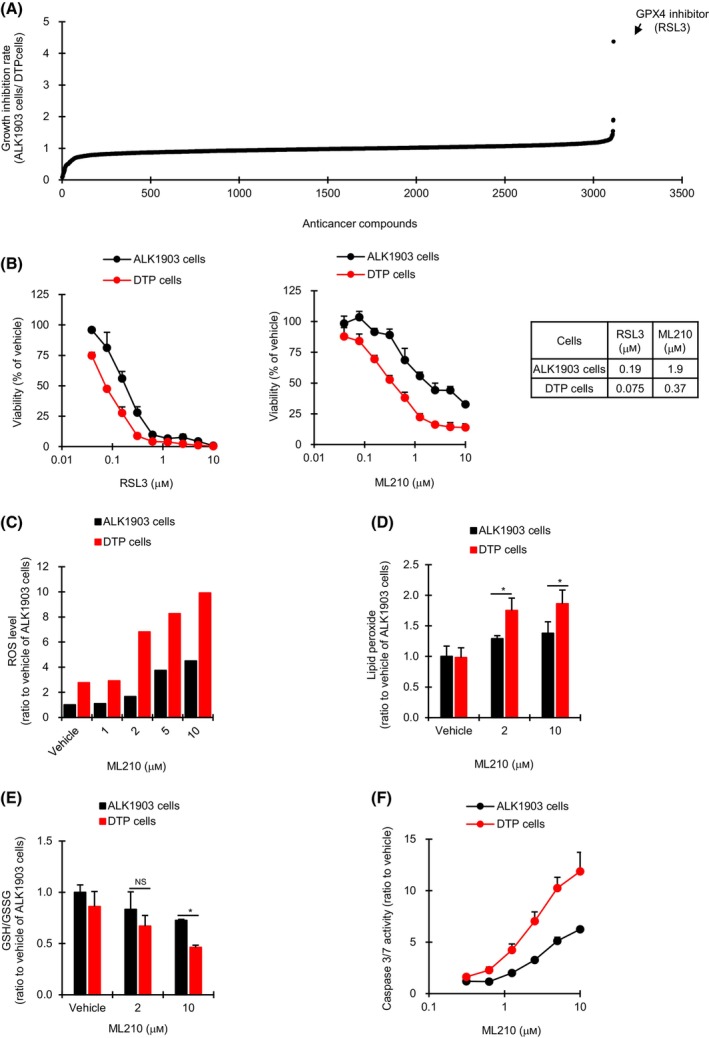
Effect of GPX4 inhibitors on ALK1903 and DTP cells. (A) Anticancer compound library screening with ALK1903 and DTP (drug‐tolerant persister) cells. Among the 3114 compounds in the library, the top compound with the strongest antiproliferative effect on DTP cells relative to ALK1903 cells was a GPX4 inhibitor (*n* = 2), indicated here with an arrow. (B) Cells were cultured with RSL3 or ML210 for 6 days (*n* = 3). The IC_50_ values are shown. (C–E) Cells were cultured with ML210 for 1 h (C), 3 h (D), or 6 h (E), and the amount of cellular ROS (*n* = 2) (C), lipid peroxide (*n* = 3) (D) or GSH/GSSG (*n* = 3) (E) was measured, respectively. *P < 0.05 between ALK1903 cells and DTP cells in the Student's *t*‐test by the Holm method. NS, not significant. (F) ALK1903 and DTP cells were cultured with ML210 for 2 days, and caspase 3/7 activities were measured (*n* = 3). Values were shown as mean ± SD (standard deviation). Data are representative of two independent experiments.

To explore the mechanisms underlying the emergence of DTP cells, we screened a library of anticancer compounds with ALK1903 and its DTP cells. To identify novel candidates specifically required for survival of cells subjected to alectinib treatment, we calculated each compound's antiproliferative effect on DTP cells as a ratio of its antiproliferative effect on parental ALK1903 cells. Out of the 3114 library compounds, an inhibitor of GPX4 (RSL3) showed the strongest inhibitory effect on DTP cells relative to ALK1903 cells (Fig. 2A). To confirm the results of this screening, a cell proliferation assay was performed using GPX4 inhibitors RSL3 and ML210. These two GPX4 inhibitors showed a stronger antiproliferative effect on DTP cells than on ALK1903 cells (Fig. 2B), indicating that GPX4 is involved in alectinib‐induced DTP state. After 6 days of exposure in ALK1903 cells, alectinib inhibited the growth of the parental cells by approximately 32.6% at the highest concentration of 1000 nm (Fig. 1C), whereas the GPX4 inhibitor (RSL3 and ML210) at the highest concentration of 10 mM inhibited the growth by approximately 99.2% and 67.4%, respectively (Fig. 2B). Therefore, under the *in vitro* 6‐day exposure condition in ALK1903 cells, a GPX4 inhibitor at a high concentration would be a useful treatment option with more potent growth inhibitory effects than alectinib, not only on DTP cells but also on ALK1903 parental cells.

To examine the mechanisms though which DTP cells depend on GPX4, we analyzed the levels of cellular ROS and lipid peroxide and the GSH/GSSG ratios with or without GPX4 inhibitors. In ALK1903 DTP cells, ML210 enhanced ROS and lipid peroxide levels and significantly decreased GSH/GSSG ratio compared to that in ALK1903 cells (Fig. 2C–E). Furthermore, apoptosis and apoptotic DNA fragmentation were increased in DTP cells in the presence of ML210 (Fig. 2F; Fig. S2A).

**Fig. 3:**
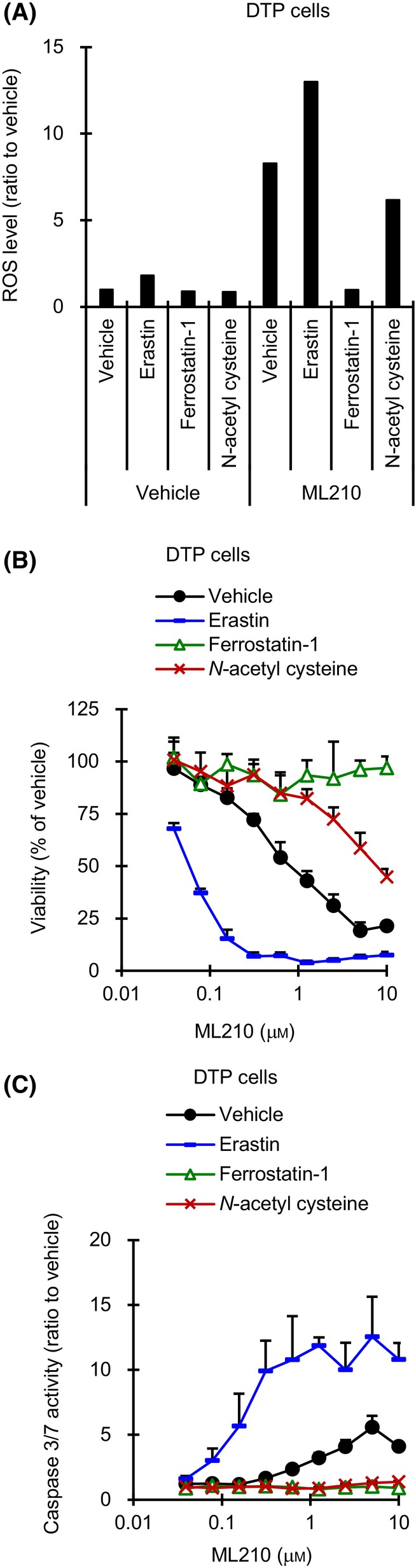
Effect of GPX4 inhibitor plus ferroptosis inducer or ferroptosis inhibitors on ALK1903 and DTP cells. (A) DTP (drug‐tolerant persister) cells were cultured in the presence of 2 μm erastin, 2 μm ferrostatin‐1, or 5 mm *N*‐acetyl cysteine with or without 2 μm ML210 for 6 h and ROS level was measured (*n* = 2). (B) DTP cells were treated with indicated inhibitors for 6 days and viability was measured (*n* = 3). (C) DTP cells were treated with indicated inhibitors for 2 days and caspase 3/7 activities were measured (*n* = 3). Values were shown as mean ± SD (standard deviation). Data are representative of two independent experiments.

To confirm whether the cell growth inhibition is due to ferroptosis and apoptosis occurring as a result of ML210‐induced ROS accumulation in DTP cells, we treated DTP cells with a ferroptosis inducer (erastin) and two ferroptosis inhibitors (ferrostatin‐1 and N‐acetyl cysteine). Erastin leads to inactivation of GPX4 protein via depleting GPX4 cofactor GSH, leading to an increase in the amount of intracellular ROS [16]. Ferrostatin‐1 attenuates the accumulation of lipid ROS in the cell membrane by trapping radical antioxidants [17], and N‐acetyl cysteine scavenges intracellular ROS through increasing intracellular GSH levels and its thiol‐disulfide exchange activity [18]. Compared to treatment with ML210 alone, co‐treatment of DTP cells with ML210 plus erastin enhanced ROS level approximately 2 fold and increased apoptosis induction and cell growth inhibition (Fig. 3A–C). In contrast, co‐treatment of DTP cells with ML210 plus ferrostatin‐1 or N‐acetyl cysteine tended to decrease ROS level compared to treatment with ML210 alone and recovered cells from ML210‐induced apoptosis and cell death (Fig. 3A–C). Thus, ML210‐induced cell death via ferroptosis and apoptosis was enhanced through increasing ROS accumulation in DTP cells, suggesting that GPX4 may be activated to reduce cellular ROS levels under alectinib treatment. If this is indeed the case, it means that activated GPX4 would promote evasion from alectinib‐induced cell death in DTP cells via decreasing the ROS‐mediated oxidative damage involved in ferroptosis and apoptosis.

### 3.3 DTP cells evade ALK‐TKI‐induced ROS‐mediated cell death through GPX4 activity

**Fig. 4:**
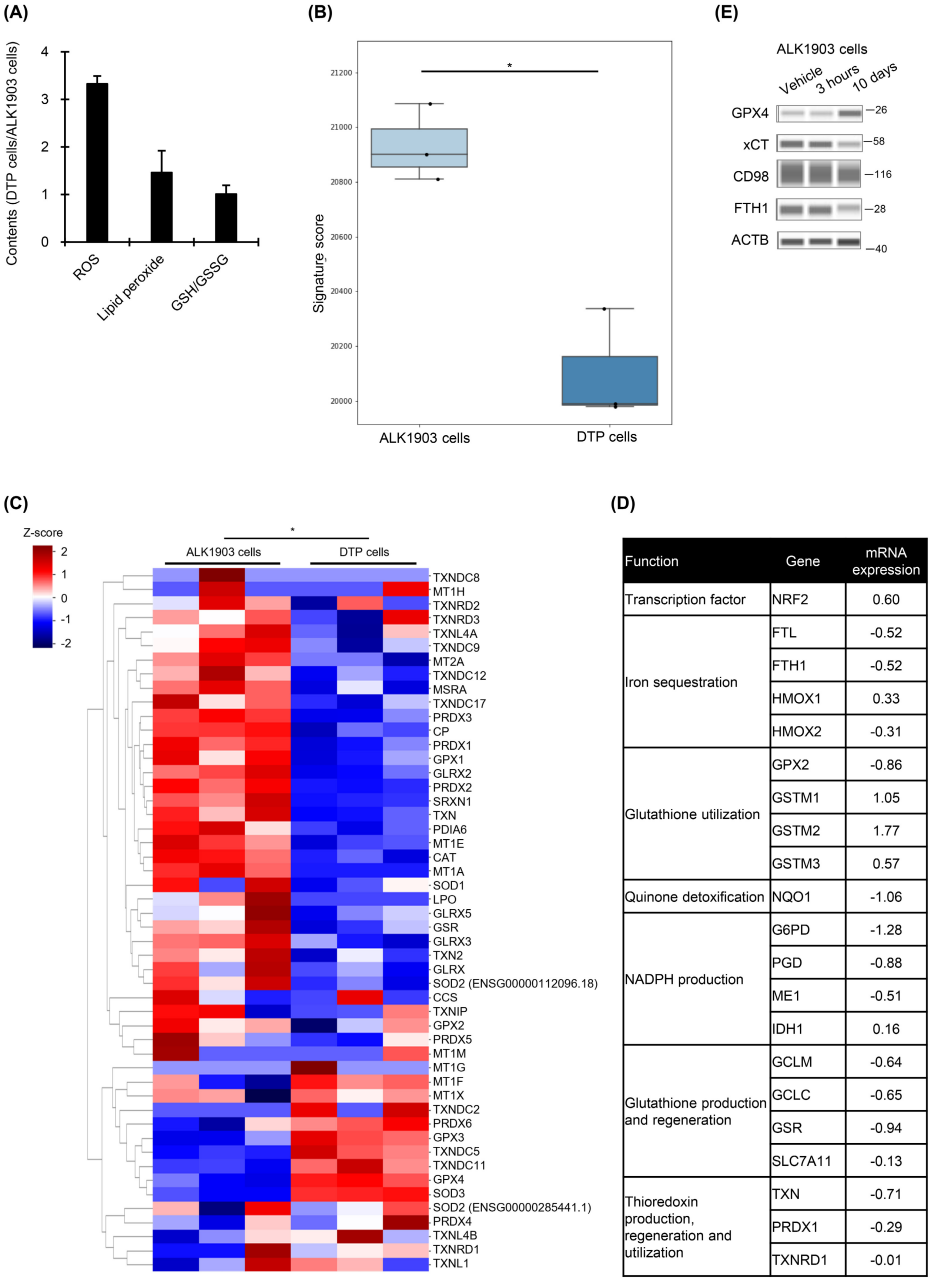
Level of antioxidant gene expression in ALK1903 and DTP cells. (A) The levels of ROS, lipid peroxide, and GSH/GSSG in DTP cells relative to levels in ALK1903 cells were measured (*n* = 3). The mRNA expressions related to antioxidant activity in DTP cells relative to ALK1903 cells was determined by ssGSEA analysis (B) and heatmap analysis (C) using RNA sequence (*n* = 3). *P < 0.05 between ALK1903 cells and DTP (drug‐tolerant persister) cells in the Welch's t‐test (B) and a two‐tailed Wilcoxon rank‐sum test (C). (D) The mRNA levels of NRF2 target genes in DTP cells relative to levels in ALK1903 cells were analyzed by RNA‐seq (*n* = 3). (E) Immunoblots of cell lysates from ALK1903 cells treated with 1000 nm alectinib for 3 h or 10 days. Values were shown as mean ± SD (standard deviation). Data are representative of two independent experiments.

To analyze the mechanisms underlying why DTP cells are sensitive to ML210 treatment, we measured the levels of ROS and lipid peroxide and the GSH/GSSG ratio. Although ROS level was elevated approximately 3 fold in DTP cells as compared to in ALK1903 cells, changes in the other two factors were limited (Fig. 4A). Both ssGSEA analysis and heatmap analysis showed a significant downregulation of mRNA expressions directly related to antioxidant activity [19] in DTP cells as compared to ALK1903 cells (Fig. 4B,C). Further, the mRNA expression of 15 out of 20 antioxidant genes regulated by NRF2, a key redox‐sensitive transcription factor [20], was also decreased in DTP cells (Fig. 4D). Since the amino acid antiporter xCT sustains the production of GSH through serial reactions after exchanging extracellular cystine for intracellular glutamate, the synthesis of GSH depends on the availability of cellular cysteine whose level is regulated by xCT activity [21]. We found that xCT and FTH1 protein levels in ALK1903 cells were decreased 10 days after treatment with alectinib as compared with levels before treatment, whereas GPX4 protein level was increased (Fig. 4E).

From these data showing elevated levels of ROS that arise through decreased levels of various antioxidant factors and decreased GSH synthesis, it might be expected that ROS‐mediated cell death should occur in alectinib‐induced DTP cells. However, DTP cells concurrently upregulated GPX4 protein, suggesting that ALK1903 DTP cells are able to evade ROS‐mediated cell death by reducing ROS level in a GPX4‐dependent manner.

### 3.4 DTP cells evade ALK‐TKI–induced cell death through activation of EGFR and HER3 signaling

**Fig. 5:**
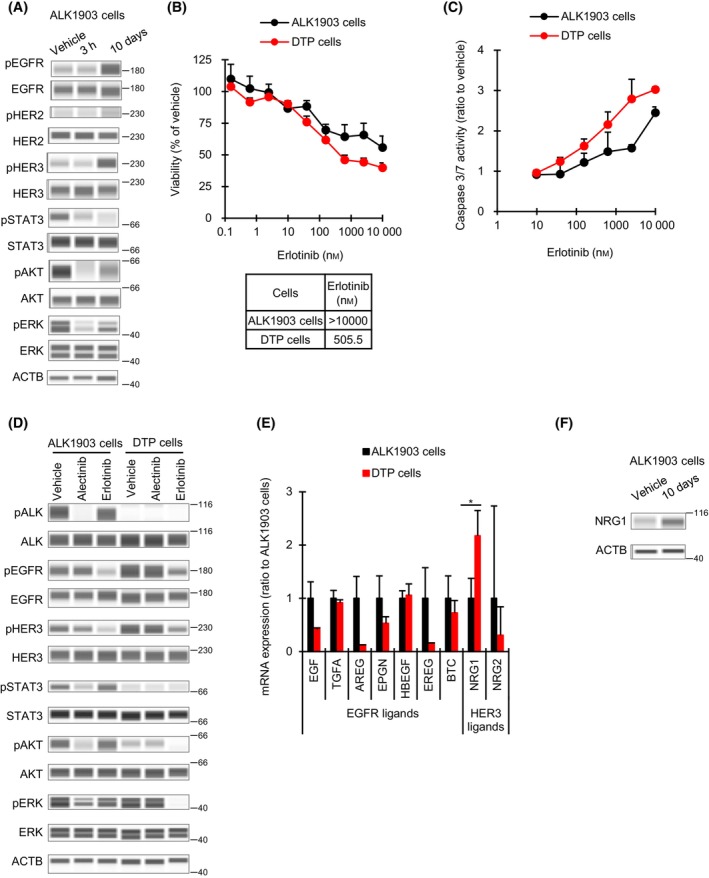
Effect of EGFR‐TKI on ALK1903 and DTP cells. (A) Immunoblots of cell lysates from ALK1903 cells treated with 1000 nm alectinib for 3 h or 10 days (*n* = 1). The data represented an image from two independent experiments. (B, C) Cells were cultured with erlotinib for 6 days (*n* = 3) (B) or 2 days (*n* = 3) (C), and the viability or the caspase 3/7 activities were measured, respectively. IC_50_ values are shown. (D) Immunoblots of cell lysates from ALK1903 and DTP (drug‐tolerant persister) cells treated with 1000 nm alectinib or 1000 nm erlotinib for 3 h (*n* = 1). The data represented an image from two independent experiments. (E) Levels of mRNA expression of ligands of EGFR and HER3 in DTP cells relative to levels in ALK1903 cells was analyzed by RNA‐seq (*n* = 3). *P < 0.05 between ALK1903 cells and DTP cells in the Student's *t*‐test. (F) Immunoblots of cell lysates from ALK1903 cells treated with 1000 nm alectinib for 10 days (*n* = 1). The data represented an image from two independent experiments. Values were shown as mean ± SD (standard deviation). Data are representative of two independent experiments.

It has been recently reported that HER3 activation also contributes to the emergence of ALK‐TKI–induced DTP cells in *ALK* ^+^ NSCLC cells [22]. To assess the contribution of ERBB family members, including EGFR, HER2, and HER3, to cells acquiring DTP states under alectinib treatment, we measured their phosphorylation levels in ALK1903 DTP cells. Although ALK phosphorylation was completely suppressed from 3 h to 10 days during alectinib treatment (Fig. 1E), the phosphorylation levels of EGFR and HER3, as well as AKT and ERK which are involved in ERBB downstream signaling, were greater at 10 days than at 3 h (Fig. 5A). Furthermore, DTP cells were slightly more sensitive to the EGFR‐TKI erlotinib and showed increased erlotinib‐induced apoptosis compared to ALK1903 cells (Fig. 5B,C). Although alectinib inhibited phosphorylation of AKT and ERK in ALK1903 cells, their phosphorylations were not inhibited by alectinib in DTP cells despite the complete suppression of ALK phosphorylation (Fig. 5D). In contrast, erlotinib did not inhibit phosphorylation of AKT or ERK in ALK1903 cells, whereas erlotinib inhibited phosphorylation of EGFR, HER3, AKT, and ERK in DTP cells (Fig. 5D). Among all ligands of EGFR and HER3, mRNA expression of neuregulin 1 (NRG1) was significantly increased in DTP cells as compared to in ALK1903 cells (Fig. 5E). Moreover, DTP cells expressed high levels of NRG1 protein compared to ALK1903 cells (Fig. 5F). Therefore, in addition to GPX4, activation of the EGFR and HER3 by upregulation of NRG1 may also be important for survival of DTP cells under alectinib treatment.

### 3.5 Combined ALK‐TKI plus EGFR‐TKI plus GPX4 inhibitor suppresses growth of ALK1903 cells

**Fig. 6:**
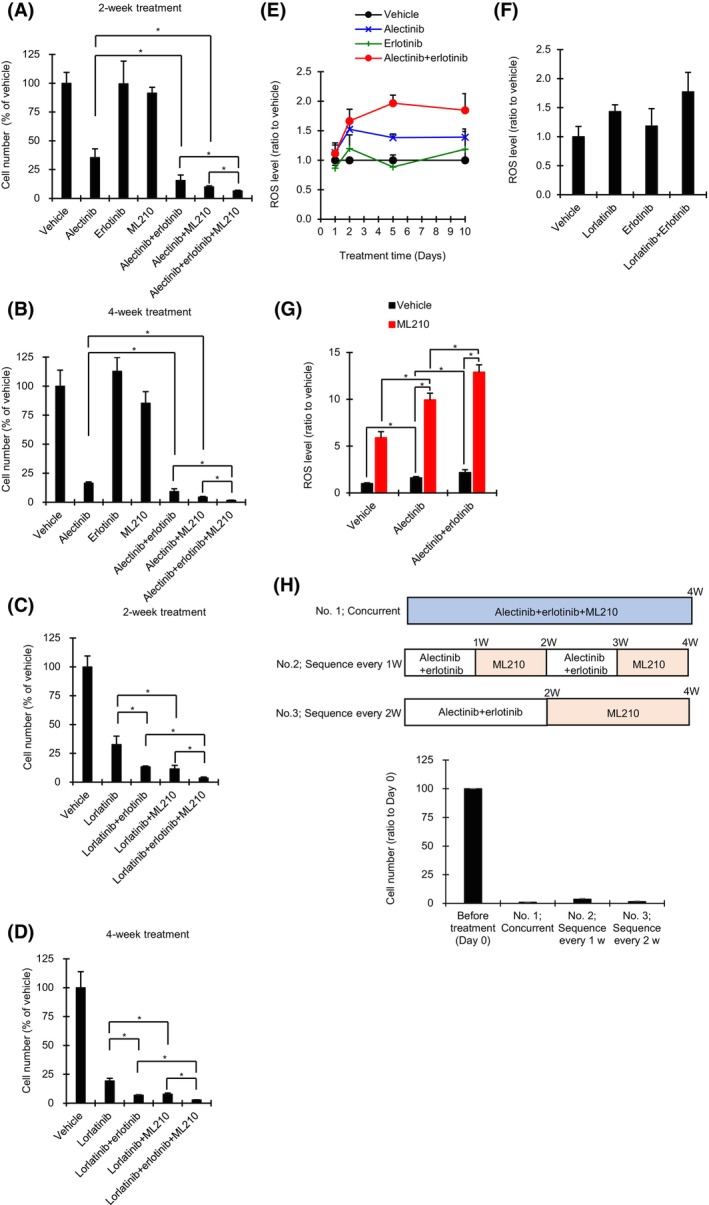
Effects of combination of ALK‐TKIs, ML210, and erlotinib on ALK1903 cells. (A–D) Cells were cultured with 100 nm erlotinib, 1 μm ML210, and 1000 nm alectinib (A and B) or 1000 nm lorlatinib (C and D) for 2 weeks (A and C) or 4 weeks (B and D), respectively. The numbers of drug‐treated cells as a percentage of vehicle‐treated cells were measured with an NC‐3000 cell counter (*n* = 3). *P < 0.05 between the two treatments linked by line in the Student's *t*‐test by the Holm method. (E) Cells were cultured with a combination of 100 nm erlotinib plus 1000 nm alectinib, or with each single agent for 10 days. After 1, 2, 5, and 10 days, the ROS levels of drug‐treated cells relative to those of vehicle‐treated cells were measured (*n* = 3). (F) Cells were cultured with a combination of 100 nm erlotinib plus 1000 nm lorlatinib, or with each single agent for 10 days (*n* = 3). (G) Cells were cultured in the presence of 1000 nm alectinib with or without 100 nm erlotinib for 6 days. After washing the cells, cells were cultured in the presence of 1 μm ML210 for 1 h (*n* = 3). *P < 0.05 between the two treatments linked by lines in the Student's *t*‐test by the Holm method. SD, standard deviation. (H) Cells were cultured in three types of treatment conditions for 4 weeks; (1) concurrent combination with 1000 nm alectinib, 100 nm erlotinib and 1 μm ML210, (2) sequential treatment every 1 week of combination of 1000 nm alectinib and 100 nm erlotinib followed by 1 μm ML210, (3) sequential treatment every 2 weeks of combination of 1000 nm alectinib and 100 nm erlotinib followed by 1 μm ML210. Cell numbers were measured by a cell counter (*n* = 3). Values were shown as mean ± SD (standard deviation). Data are representative of two independent experiments.

We next assessed whether GPX4 inhibition in combination with ALK‐ and/or EGFR‐TKIs suppresses proliferation of ALK1903 cells. Although ALK1903 cells were mostly insensitive to erlotinib or ML210 alone (ratio of drug‐treated to vehicle‐treated viable cell numbers, > 85%), alectinib‐induced cell growth inhibition was significantly enhanced upon co‐treatment with erlotinib or ML210 compared to with alectinib alone for 2 or 4 weeks (Fig. 6A,B). Furthermore, triple combination treatment with alectinib plus erlotinib plus ML210 significantly inhibited cell proliferation compared to each double combination (Fig. 6A,B). Similar to the effects of alectinib treatment, triple combination with lorlatinib plus erlotinib plus ML210 also induced significant cell growth inhibition compared to each double combination (Fig. 6C,D).

To analyze the mechanisms underlying these triple combination effects, we measured the time course of ROS levels. Under co‐treatment with alectinib plus erlotinib, the amount of ROS tended to be higher than with each agent alone after 5 days (Fig. 6E), and under double combination treatment with lorlatinib plus erlotinib, ROS level tended to be higher than with each agent alone after 10 days (Fig. 6F). ROS levels were significantly enhanced after 6 days of alectinib treatment, and the combination of erlotinib plus alectinib induced further significant enhancement of ROS levels compared to treatment with alectinib alone (Fig. 6G). Notably, addition of ML210 significantly increased alectinib‐induced ROS accumulations with or without erlotinib (Fig. 6G).

We further evaluated the effects of this triple combination over 4 weeks, using three different treatment sequences: (No. 1) concurrent treatment, comprising one 4‐week cycle of alectinib plus erlotinib plus ML210; (No. 2) a sequential treatment comprising 2 cycles each of 1 week of alectinib plus erlotinib followed by 1 week of ML210; and (No. 3) a sequential treatment comprising 1 cycle of 2 weeks of alectinib plus erlotinib followed by 2 weeks of ML210. The percentages of viable cell numbers remaining at the conclusion of concurrent treatment (No. 1), sequence treatments No. 2 and No. 3 were 1.0%, 3.6%, and 1.6%, respectively (Fig. 6H), indicating that both concurrent combination and sequential treatment regimens have potential to suppress the growth of ALK1903 cells to at least <4.0% of that before the start of treatment.

### 3.6 Triple combined treatment with alectinib plus FGFR inhibitor plus GPX4 inhibitor suppresses growth of NCI‐H2228 cells

**Fig. 7:**
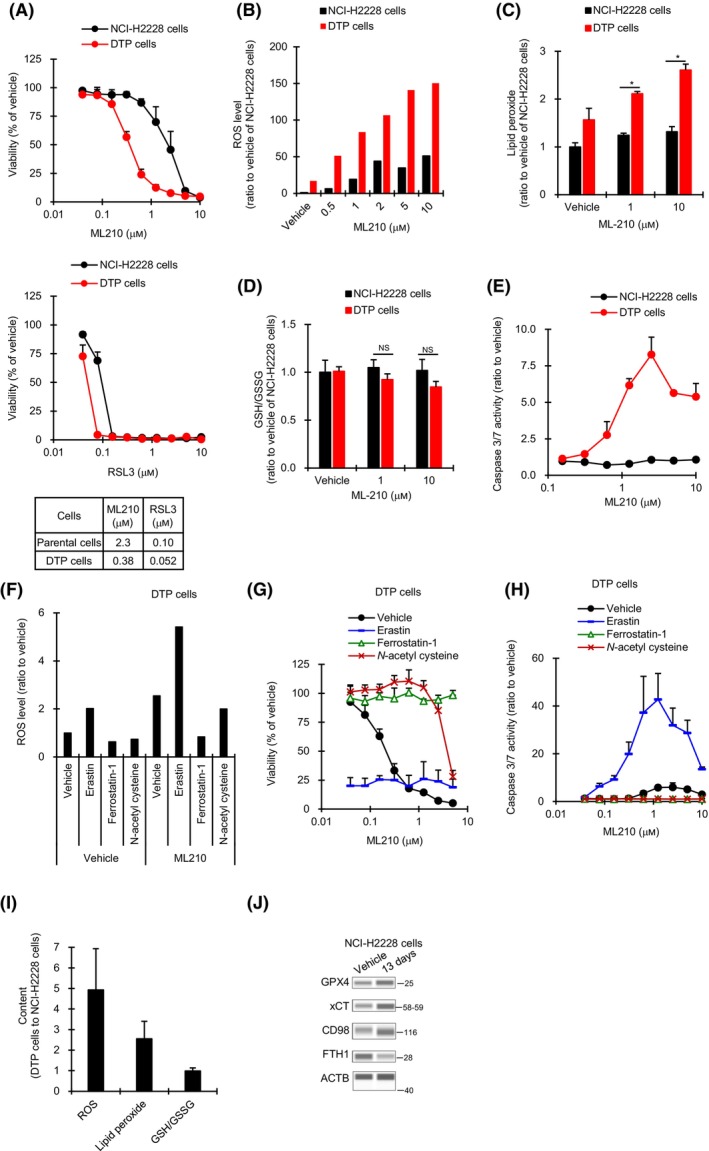
Effect of GPX4 inhibitors on NCI‐H2228 and DTP cells. (A) Cells were cultured with RSL3 or ML210 for 6 days (*n* = 3). IC_50_ values are shown. (B–D) Cells were cultured with ML210 for 1 h (*n* = 2) (B), 4 h (*n* = 3) (C), or 6 h (*n* = 3) (D), and the amount of cellular ROS (B), lipid peroxide (C), or GSH/GSSG (D) was measured, respectively. *P < 0.05 between NCI‐H2228 cells and DTP (drug‐tolerant persister) cells in the Student's *t*‐test by the Holm method. NS, not significant. (E) Cells were cultured with ML210 for 2 days, and caspase 3/7 activities were measured (*n* = 3). (F–H) DTP cells were cultured in the presence of 2 μm erastin, 2 μm ferrostatin‐1, or 5 mm *N*‐acetyl cysteine with or without 1 μm ML210 for 1 day (*n* = 2) (F), 6 days (*n* = 3) (G), or 2 days (*n* = 3) (H), and ROS level, viability, or apoptosis induction was measured, respectively. (I) Levels of ROS, lipid peroxide, or GSH/GSSG of DTP cells relative to NCI‐H2228 cells (*n* = 3). (J) Immunoblots of cell lysates from NCI‐H2228 cells treated with 1000 nm alectinib for 13 days (*n* = 1). The data represented an image from two independent experiments. Values were shown as mean ± SD (standard deviation). Data are representative of two independent experiments.

We next used NCI‐H2228 cells to assess whether the survival of alectinib‐induced DTP cells also depends on GPX4 in other types of *ALK* ^+^ NSCLC cells. Similar to the results in ALK1903 cells, sensitivity to both ML210 and RSL3 was increased in alectinib‐induced NCI‐H2228 DTP cells generated by 13 days 1000 nm of alectinib treatment (Fig. 7A), and ML210 treatment enhanced the levels of ROS, lipid peroxide, and apoptosis induction in the DTP cells, and tended to reduce the ratio of GSH/GSSG (Fig. 7B–E). Furthermore, ML210‐induced DNA fragmentation was observed in NCI‐H2228 DTP cells (Fig. S2B). Analyses using erastin, N‐acetyl cysteine, and ferrostatin‐1 with ML210 revealed that ML210‐induced ferroptosis and apoptosis were regulated by the amount of ROS in DTP cells (Fig. 7F–H). In DTP cells, ROS levels were elevated approximately 5 fold, and lipid peroxidase tended to be increased as compared to in parental NCI‐H2228 cells (Fig. 7I). FTH1 protein level was decreased, whereas GPX4 protein level was increased in DTP cells compared to NCI‐H2228 cells (Fig. 7J), suggesting that the GPX4 dependency may be a common feature of alectinib‐induced DTP cells.

**Fig. 8:**
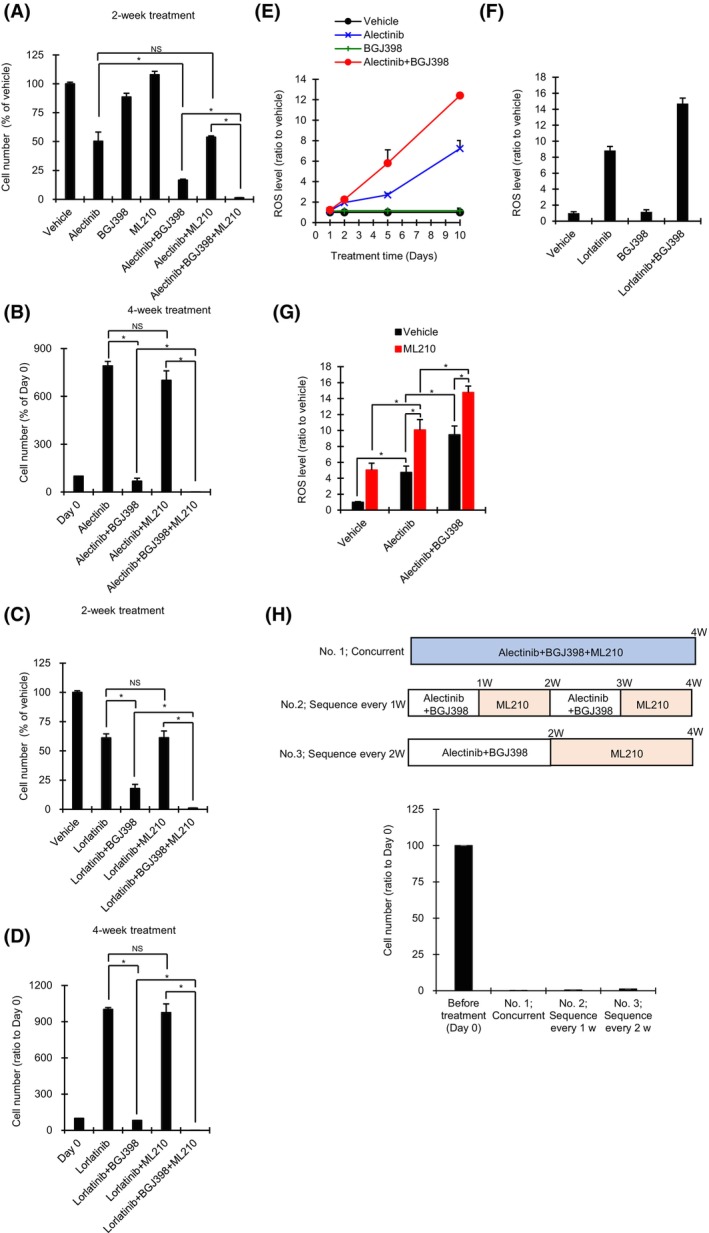
Effects of combination treatment with ALK‐TKIs, ML210, and BGJ398 on NCI‐H2228 cells. (A–D) Cells were cultured with 300 nm BGJ398, 1 μm ML210, and 1000 nm alectinib (A and B) or 1000 nm lorlatinib (C and D) for 2 weeks (A and C) or 4 weeks (B and D) (*n* = 3), respectively. *P < 0.05 between the two treatments linked by line in the Student's *t*‐test by the Holm method. NS, not significant. (E) Cells were cultured with a combination of 300 nm BGJ398 plus 1000 nm alectinib, or with each single agent for 10 days. After 1, 2, 5, and 10 days, the ROS levels of drug‐treated cells relative to those of vehicle‐treated cells were measured (*n* = 3). (F) Cells were cultured with a combination of 300 nm BGJ398 plus 1000 nm lorlatinib, or with each single agent for 10 days (*n* = 3). (G) Cells were cultured in the presence of 1000 nm alectinib with or without 300 nm BGJ398 for 6 days. After washing the cells, cells were cultured in the presence of 1 μm ML210 for 1 h (*n* = 3). *P < 0.05 between the two treatments linked by lines in the Student's *t*‐test by the Holm method. (H) Cells were cultured in three types of treatment conditions for 4 weeks; (1) concurrent combination with 1000 nm alectinib, 300 nm BGJ398 and 1 μm ML210, (2) sequential treatment every 1 week of combination of 1000 nm alectinib and 300 nm BGJ398 followed by 1 μm ML210, (3) sequential treatment every 2 weeks of combination of 1000 nm alectinib and 300 nm BGJ398 followed by 1 μm ML210. Cell numbers were measured by a cell counter (*n* = 3). Values were shown as mean ± SD (standard deviation). Data are representative of two independent experiments.

On the other hand, in a separate study, we found that NCI‐H2228 DTP cells can evade alectinib‐induced cell death through activation of a bypass signaling pathway with FGFR1, and that the combination of an FGFR‐TKI (BGJ398) plus alectinib enhanced inhibition of NCI‐H2228 cell growth [23]. Therefore, we assessed the effect of the triple combination of alectinib plus BGJ398 plus ML210 on NCI‐H2228 cells. Similar to the results with ALK1903 cells, growth of NCI‐H2228 cells was significantly inhibited by 2‐ or 4‐week treatment with ALK‐TKIs plus BGJ398, and this inhibition of cell growth was further significantly enhanced by inclusion of ML210 (Fig. 8A–D). ROS levels in NCI‐H2228 cells treated for 10 days with the double combination of ALK‐TKI (alectinib or lorlatinib) plus FGFR1 inhibitor (BGJ398) tended to be greater than levels in cells treated with each single agent alone (Fig. 8E,F). Moreover, ROS levels were significantly enhanced by 6 days of treatment with alectinib, and these levels were further significantly increased with the combination of alectinib plus BGJ398; inclusion of ML210 significantly increased alectinib‐induced ROS accumulation with or without BGJ398 (Fig. 8G).

We evaluated the effects of this triple combination treatment over 4 weeks, using three different treatment sequences. The two sequential treatments (one comprising 2 cycles each of 1 week of alectinib plus BGJ398 followed by 1 week of ML210 (No. 2), the other comprising one cycle of 2 weeks of alectinib plus BGJ398 followed by 2 weeks of ML210 (No. 3)) showed almost the same inhibitory effects as 4 weeks of concurrent treatment with the combination of alectinib plus BGJ398 plus ML210 (No. 1) (for each treatment sequence, the percentage of cells remaining after 4 weeks was < 2.0%) (Fig. 8H).

These findings in ALK1903 and NCI‐H2228 cells suggest that GPX4 inhibitors would suppress the generation of alectinib‐induced DTP cells, and the efficacy of GPX4 inhibitors might be further increased when combined with activated bypass signal inhibitors, such as EGFR‐TKIs or FGFR‐TKIs.

## 4 Discussion

Although genotype‐directed targeted therapy is the standard of care and elicits a dramatic response in many cancers, these therapies are rarely curative. In *ALK* ^+^ NSCLC, a complete response is observed in fewer than 5% of patients who receive ALK‐TKIs [24]. One reason for this discouraging result is that a small number of residual DTP cells can survive initial ALK‐TKI exposure and develop drug resistance during treatment. Therefore, it is essential to establish novel effective and low‐toxicity DTP‐targeting therapies to overcome this resistance.

*In vitro* studies with *ALK* ^+^ NSCLC cells have identified several diverse types of ALK‐TKI–induced DTP cells, including those including those in which YAP‐TEAD, HER3, or glycogen synthase kinase 3 (GSK3) pathways are activated [5, 22, 25, 26]. Although it is not clear whether therapies targeting these pathways would lead to emergence of yet other types of DTP cell, it is important to develop novel combination therapies that can enhance the efficacy of any DTP‐targeted therapy.

Here, using alectinib‐induced DTP cells generated from two different *ALK* ^+^ NSCLC cells, we demonstrated that although both DTP cells contained high amounts of ROS due to the downregulation of antioxidant genes by ALK signal inhibition under alectinib treatment, both DTP cells were able to evade or minimize ROS‐induced cell death including ferroptosis and apoptosis by erasing ROS through increasing GPX4 protein levels. Similar to these findings with *ALK* ^+^ NSCLC cells, it is reported that in DTP cells in EGFR^+^ NSCLC, HER2^+^ breast cancer, and BRAF^+^ melanoma, the gene signatures of ROS production are increased after treatment with clinically relevant TKIs and the expression of antioxidant genes is also decreased [27]. Thus, the DTP state induced by oncogenic kinase inhibition appears to share similar features of ROS enhancement as well as downregulation of antioxidant genes across cancer cells from different oncogenic mutations and tumor tissues.

When we cultured DTP cells with alectinib‐free medium for 15 days, ALK phosphorylation was restored compared to those cultured for 1 day (Fig. S3). Although alectinib is not present the culture medium under both conditions, the mechanism of the difference in ALK phosphorylation is unknown. Since alectinib has a high human plasma protein binding rate of ≥ 99% [28], it is possible that < 1% free alectinib is passively distributed in DTP cells and then bound to various intracellular proteins. This suggests that the phosphorylation of ALK is suppressed by residual alectinib after culturing DTP cells for 1 day in the absence of alectinib, but ALK phosphorylation is restored after culturing for 15 days due to markedly decreased intracellular alectinib. Further studies are needed to clarify our hypothesis. We used phospho‐STAT3 antibody #9145 as cell signaling technology to detect Tyr705. At 3 h and 10 days after 1000 nm alectinib exposure, the phosphorylation of STAT3 (Tyr705) was markedly reduced, but slight phosphorylation was detected, as shown in Fig. 5A. Furthermore, a slight level of Tyr705 of STAT3 phosphorylation was also detected in DTP cells treated with vehicle for 3 h (Fig. 5D). Therefore, we believe that the activity of STAT3 is slightly maintained, although that of STAT3 phosphorylation (Tyr705) is considerably decreased in DTP cells.

The antioxidant protein, FTH1 functions to store free iron and reduce iron‐mediated ROS production, and its overexpression suppresses erastin‐induced ferroptosis and ROS upregulation in neuroblastoma cells [29]. In this study, one of the mechanisms underlying alectinib‐induced ROS enhancement in DTP cells may be caused by downregulation of FTH1 protein, because FTH1 protein was downregulated in each of the two types of DTP cells. Three major transcription factors—activator protein 1 (AP‐1), NFκB, and NRF2—participate in the expression of various antioxidant genes [30, 31, 32]. Of these, AP‐1, which is a complex of c‐FOS and c‐JUN, is especially important for the antioxidant response because it can enhance the transcriptional activity of NFκB as well as NRF2 [33, 34]. Phosphorylation of AP‐1 is regulated by c‐Jun N‐terminal kinase (JNK) [35]. Furthermore, activated ERK itself translocates to the nucleus to activate not only transcription of c‐JUN and c‐FOS genes but also phosphorylation of c‐FOS and c‐JUN followed by AP‐1 transcriptional activation [36]. In *NPM*‐*ALK* ^+^ anaplastic large‐cell lymphoma (ALCL) cells, NPM‐ALK kinase phosphorylates and activates JNK, and the activated JNK subsequently phosphorylates and activates c‐Jun followed by AP‐1 transcriptional activation [37]. Since the EML4‐ALK in *ALK* ^+^ NSCLC cells has an ALK kinase domain fragment identical to that in NPM‐ALK [38], the EML4‐ALK is thought to be similarly responsible for AP‐1 transcriptional activity, suggesting that decreased AP‐1 activity resulting from ALK‐TKI treatment in *ALK* ^+^ NSCLC cells would lead to downregulation of various antioxidant gene expressions and enhancement of cellular ROS levels.

Although further studies are needed to assess this hypothesis, the *in vitro* DTP features of TKI‐induced ROS upregulation described above have also been observed in residual tumor tissue specimens from *in vivo* mouse models of EGFR^+^ lung cancer after EGFR‐TKI treatment [27]. Furthermore, upregulated of ROS related signatures are observed in residual tumors from TKI‐treated patients with EGFR^+^ NSCLC, HER2^+^ breast cancer, and BRAF^+^ melanoma [27], suggesting that the findings of our study with *ALK* ^+^ NSCLC cells may also possibly be observed in patients with *ALK* ^+^ NSCLC. We found that alectinib‐induced DTP cells survived through activation of EGFR/HER3 with NRG1 or through activation of FGFR1 with FGF2 ligand [23], and that co‐treatment with EGFR‐TKI or FGFR‐TKI plus ALK‐TKIs increased ROS levels compared to each single agent alone. Since both JNK and ERK are downstream signaling molecules of ALK, EGFR, and FGFR, combination treatment with EGFR‐TKI or FGFR‐TKI plus an ALK‐TKI would strongly inhibit transcription as well as activation of AP‐1 via inhibition of JNK and ERK, which would lead to strongly enhanced ROS levels compared to each agent alone, highlighting the importance of double combination treatments with EGFR‐ or FGFR‐TKI plus ALK‐TKI to increase ROS‐induced cell death over single agents.

Further, it is also reported that cancer‐associated fibroblasts (CAFs) play a role in the emergence of DTP cells [39], and that CAF‐provided ligands and cytokines confer receptor tyrosine kinase activation, resulting in drug resistance [40]. Although we showed upregulated NRG1 and FGF2 level [23] in the cancer cells (DTP cells), it is possible that CAFs and other types of cells actively provide ERBB family and FGFR family ligands. In our model at least, results from both ALK1903 and NCI‐H2228 cells suggest that GPX4 is a key factor necessary for cells to enter a DTP state. In addition, ERBB family members in the case of ALK1903 and FGFR1 in the case of NCI‐H2228 also support the emergence of DTP cells. Although further research is needed, triple combination comprising ALK‐TKI plus GPX4 inhibitor plus a TKI corresponding to the bypass pathway would be helpful to eliminate the cancer cells and reduce the risk of DTP induction.

GPX4 is a central regulator of ferroptosis that prevents ROS accumulation and rescues cell membranes from ROS‐induced lipid peroxidation [41]. In this study, we found that GPX4 protein was upregulated in two types of alectinib‐induced DTP cells, and that cell death by ferroptosis and apoptosis was also induced by ML210 treatment in both DTP cells through enhancement of cellular ROS levels. Consistent with our results, GPX4 dependency of DTP cells induced by targeted TKI has also been found in HER2‐amplified breast cancer, EGFR‐mutated NSCLC, and BRAF‐mutated melanoma [10]. Upregulation of GPX4 mRNA expression has been observed *in vitro* in EGFR‐TKI‐induced DTP cells in EGFR^+^ NSCLC, and combination treatment with EGFR‐TKI plus GPX4 inhibitor was found to prevent growth of EGFR^+^ NSCLC cells *in vitro* [42]. Furthermore, we found that triple combined treatments with a double combination (EGFR‐ or FGFR‐TKI plus ALK‐TKI) plus ML210 decreased DTP cell survival and enhanced ROS as compared to each double combination. This suggests that GPX4 protein plays an essential role as a regulator of ROS‐induced cell death not only under treatment with ALK‐TKI alone but also under treatment with ALK‐TKI plus various types of bypass pathway inhibitor involving EGFR‐ and FGFR‐TKI in *ALK* ^+^ NSCLC cells. Considering these observations, initial triple combination treatment of *ALK* ^+^ NSCLC cells with ALK‐TKIs plus bypass inhibitors plus GPX4 inhibitors would effectively inhibit the emergence of DTP cells via several different modes of action involving suppression of proliferative signal transduction as well as induction of oxidative damage through upregulation of ROS.

Intriguingly, it has been found in EGFR^+^ NSCLC cells that a sequential treatment with EGFR‐TKIs plus trametinib or dasatinib following these TKIs plus GPX4 inhibitor has potent efficacy to inhibit cell growth as compared to treatment with these TKIs [42]. In this study, we found that sequential regimens of EGFR‐ or FGFR‐TKIs plus alectinib followed by GPX4 inhibitors alternately every 1 or 2 weeks was successful in reducing viable cell numbers with an efficacy comparable to that of parallel concurrent triple combination treatment in both of two types of *ALK* ^+^ NSCLC cells (Figs 6H and 8H). Previously, targeted monotherapy‐resistant cancer cells were more sensitive to the GPX4 inhibitors as they induce ferroptotic cell death in many cancer cell lines in various tumor tissues *in vitro* [10, 43, 44]. Furthermore, various types of GPX4 inhibitors, such as those blocking GPX4 activity, suppressing GPX4 expression, and degrading GPX4 protein, have been developed in preclinical studies [45]. However, no GPX4 inhibitors have reached clinical studies for cancer therapy in humans, since all conventional GPX4 inhibitors including RSL3 have had limited prospects for further clinical development due to their poor selectivity and pharmacokinetics [46]. ML210 used in this study was developed as a more selective GPX4 inhibitor with improved physiochemical and pharmacokinetic properties [47], but there are no reports using ML210 treatments in *in vivo* cancer models and the *in vivo* efficacy and toxicity remain unknown. Furthermore, acute renal failure and early death via ferroptosis was observed in inducible Gpx4 knockout mice [48]. Therefore, there is a possibility that GPX4 inhibition could lead to adverse events in normal organs and tissues, making them difficult for evaluate in a clinical study for cancer treatment in humans, and further preclinical studies with *in vivo* model are needed to assess the potential therapeutic triple combination treatment of the GPX4 inhibitors combined with ALK‐TKI and bypass signal inhibitors for ALK‐fusion‐positive NSCLC cells. However, we found that sequential regimens of bypass inhibitors plus alectinib followed by GPX4 inhibitors alternately every 1 or 2 weeks showed comparable efficacy to concurrent triple combinations *in vitro*. Therefore, even if side effects are observed with the triple combination in *in vivo* model, we believe that these sequence therapies could reduce toxicity without decreasing efficacy. Although this needs to be confirmed in future *in vivo* studies, administration of a GPX4 inhibitor after combination treatment with EGFR‐ or FGFR‐TKIs plus alectinib would be a reasonable rationale to enhance tolerability without compromising efficacy and would be a useful therapeutic option for overcoming acquired resistance to targeted TKI alone through inhibiting the emergence of DTP cells in *ALK* ^+^ NSCLC.

## 5 Conclusion

To our knowledge, this study is the first to report that GPX4 protein promotes tolerance to alectinib and maintains survival of DTP cells through reducing alectinib‐induced ROS upregulation. It is plausible that triple combined blockade of GPX4, ALK, and activated bypass signals may be a potent therapeutic strategy for patients with *ALK* ^+^ NSCLC to prevent the development of drug resistance, and lead to tumor eradication.

## Conflict of interest

Dr. Koh Furugaki, Dr. Takaaki Fujimura, Dr. Narumi Sakaguchi, and Dr. Shigeki Yoshiura are employees of Chugai Pharmaceutical Co., Ltd., and declare no conflict of interest. Dr. Yasutaka Watanabe declares no conflict of interest. Dr. Eisaku Miyauchi has received grants from Chugai Pharmaceutical and Eli Lilly Japan; has received honoraria from Ono Pharmaceutical, Eisai, Otsuka Pharmaceutical, Thermo Fisher Scientific, Bristol Myers Squibb, Daiichi Sankyo, Pfizer, Takeda Pharmaceutical, Chugai Pharmaceutical, Amgen, Eli Lilly Japan, Nippon Kayaku, Taiho Pharmaceutical, Sysmex, Boehringer‐Ingelheim Japan, MSD, Novartis, Kyowa Kirin, and Merck; and has participated on Advisory Boards of Chugai Pharmaceutical, Boehringer‐Ingelheim Japan, Eli Lilly Japan, Merck, Daiichi Sankyo, and Ono Pharmaceutical. Dr. Ken Uchibori has received honoraria for lectures from Ono Pharmaceutical, Takeda Pharmaceutical, Bristol Myers Squibb, Daiichi Sankyo, Thermo Fisher Scientific, Chugai Pharmaceutical, Eli Lilly, AstraZeneca, Novartis, and Merck. Dr. Hidetoshi Hayashi has received support from Guardant Health Japan; has received grants from IQVIA Services Japan, Syneos Health Clinical, EPS, Nippon Kayaku, Takeda Pharmaceutical, MSD, Amgen, Taiho Pharmaceutical, Bristol Myers Squibb, Janssen Pharmaceutical, CMIC, Pfizer R&D Japan, Labcorp Development Japan, Kobayashi Pharmaceutical, Pfizer Japan, Eisai, EP‐CRSU, Shionogi & Co., Ltd., Otsuka Pharmaceutical, GlaxoSmithKline, Sanofi, Chugai Pharmaceutical, Boehringer‐Ingelheim Japan, SRL Medisearch, PRA Health Sciences, Astellas Pharma, Ascent Development Services, and Bayer Yakuhin; and has received honoraria from Ono Pharmaceutical, Daiichi Sankyo, AstraZeneca, Chugai Pharmaceutical, Eli Lilly Japan, MSD, Pfizer Japan, Boehringer‐Ingelheim Japan, Merck Biopharma, 3H Clinical Trial, Novartis Pharma, Bristol Myers Squibb, Amgen, Sysmex, and Takeda Pharmaceutical. Dr. Ryohei Katayama has received research grants from Chugai Pharmaceutical and TOPPAN Inc.

## Author contributions

KF contributed to designing the study, performed RNA‐seq analysis performed *in vitro* experiments, supervised experiments, and contributed to writing the manuscript. TF performed experiments. RK contributed to perform RNA sequencing, designing the study, supervised experiments, and contributed to writing the manuscript. NS performed RNA sequencing analysis. SY supervised experiments. YW obtained clinical tissue samples for establishment of the patient‐derived cell line. KU, EM, and HH contributed to designing the study.

## Supporting information


**Fig. S1.** Effect of ALK inhibitors on ALK1903 cells.
**Fig. S2.** Effect of GPX4 inhibitors on DNA fragmentation of ALK1903 DTP cells and NCI‐H2228 DTP cells.
**Fig. S3.** Effect of ALK inhibitors on ALK1903 cells.
**Table S1.** List of antibody.

## Data Availability

Qualified researchers may request access to individual patient‐level data through the clinical study data request platform (https://www.clinicalstudydatarequest.com/Default.aspx). For further details on Chugai's Data Sharing Policy and how to request access to related clinical study documents, see www.chugai‐pharm.co.jp/english/profile/rd/ctds_request.html. Other all experimental data supporting the findings of this study are available within the article.
