# Supplementary material for: Combined blockade of GPX4 and activated EGFR/HER3 bypass pathways inhibits the development of ALK‐inhibitor‐induced tolerant persister cells in ALK ‐fusion‐positive lung cancer
Source: Mol Oncol. 2024 Oct 6;19(2):519–39. doi: 10.1002/1878-0261.13746 (PMC11793004; doi:10.1002/1878-0261.13746)
Supplement: Supplementary file 1 — Fig. S1. Effect of ALK inhibitors on ALK1903 cells. Fig. S2. Effect of GPX4 inhibitors on DNA fragmentation of ALK1903 DTP cells and NCI‐H2228 DTP cells. Fig. S3. Effect of ALK inhibitors on ALK1903 cells. Table S1. List of antibody. [file MOL2-19-519-s001.pdf]

Supplementary Figure 1

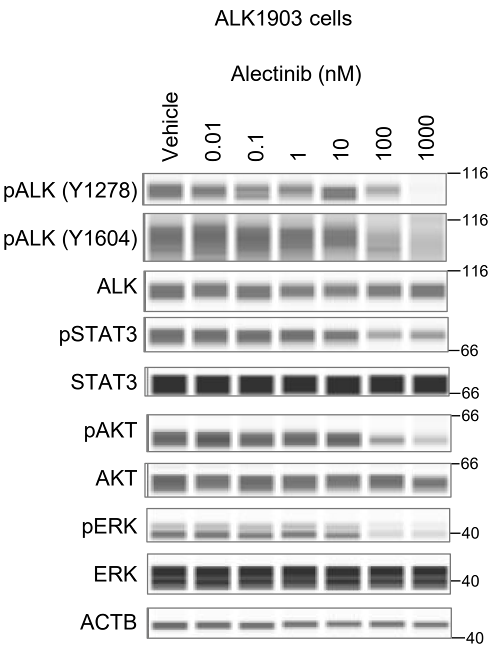

Supplementary Figure 2

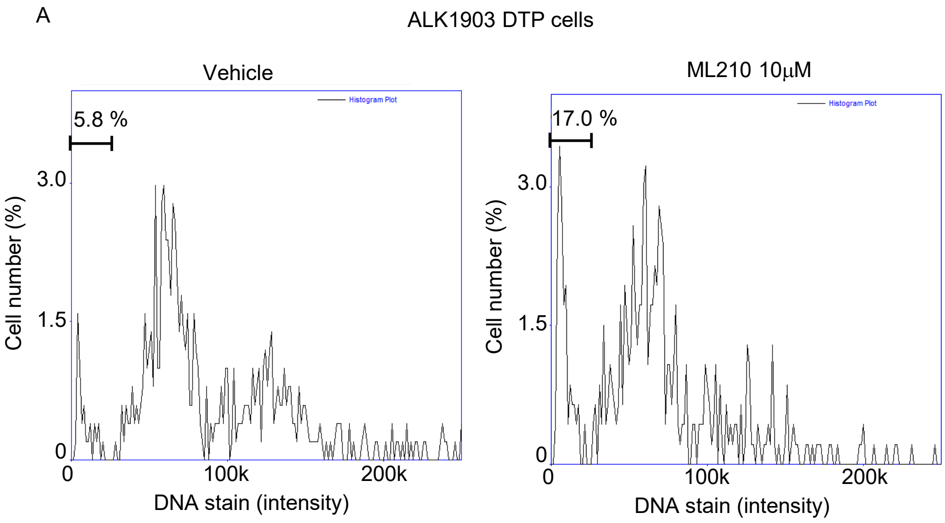

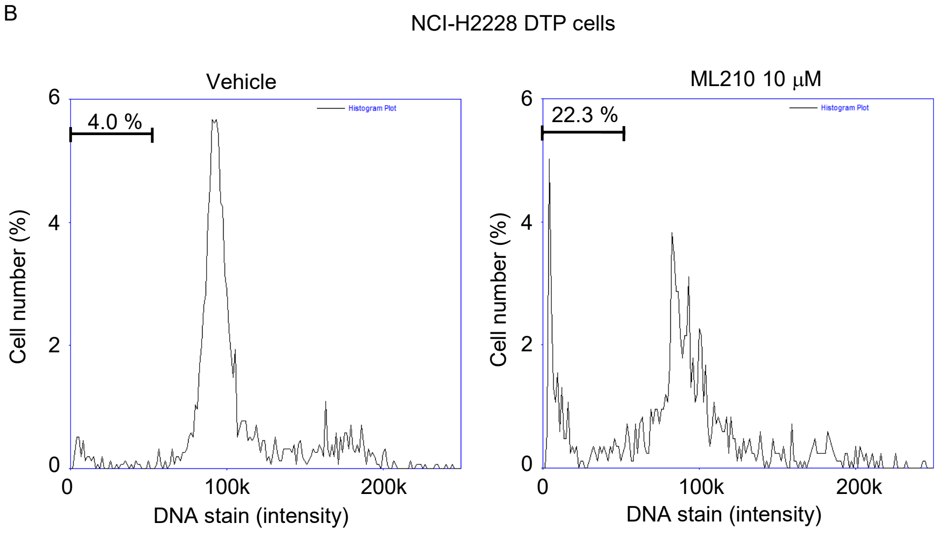

Supplementary Figure 3

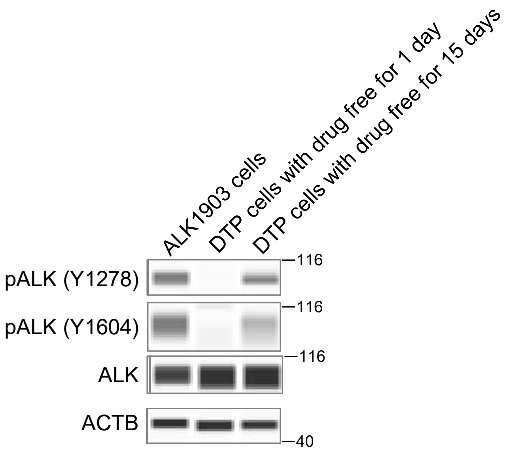

Supplementary Table 1

Antibody	Supplier	Catalog number	Phospho-site number
ALK	Cell Signaling Technology	3633	-
Phospho-ALK	Cell Signaling Technology	3341	Tyr1604
Phospho-ALK	Cell Signaling Technology	6941	Tyr1278
STAT3	Cell Signaling Technology	4904	-
Phospho-STAT3	Cell Signaling Technology	9145	Tyr705
AKT	Cell Signaling Technology	4691	-
Phospho-AKT	Cell Signaling Technology	4058	Ser473
ERK	Cell Signaling Technology	9102	-
Phospho-ERK	Cell Signaling Technology	4377	Thr202/Tyr204
β-actin	Cell Signaling Technology	4970	-
EGFR	Cell Signaling Technology	4267	-
Phospho-EGFR	Cell Signaling Technology	3777	Tyr1068
HER2	Cell Signaling Technology	3250	-
Phospho-HER2	Abcam	ab47262	Tyr877
HER3	Cell Signaling Technology	12708	-
Phospho-HER3	Cell Signaling Technology	14525	Tyr1328
CD133	Cell Signaling Technology	86781	-
CD44	Cell Signaling Technology	37259	-
BIM	Cell Signaling Technology	2933	-
GPX4	Cell Signaling Technology	52455	-
FTH1	Cell Signaling Technology	4393	-
NRG1	Cell Signaling Technology	2573	-
Cleaved PARP	Abcam	ab32064	-
xCT	Abcam	ab175186	-

The supplier, catalog number, and phospho-site number of antibody are shown.
